# Sex Differences in Patients with MASLD and Their Association with Cardiometabolic Risk Factors: Insights from the Polish Gallstone Surgery Registry

**DOI:** 10.3390/jcm14176158

**Published:** 2025-08-31

**Authors:** Iwona Gorczyca-Głowacka, Magdalena Kołomańska, Robert Mazurkiewicz, Marcin Niżnik, Krzysztof Ratnicki, Małgorzata Czerniak, Piotr Myrcha, Sebastian Lenarcik, Kryspin Mitura, Laura Kacprzak, Małgorzata Pajer, Piotr Richter, Kamil Rapacz, Maciej Sroczyński, Mateusz Szmit, Łukasz Nawacki

**Affiliations:** 1 Collegium Medicum, The Jan Kochanowski University, 25-317 Kielce, Poland; mkolomanska92@gmail.com (M.K.); rt.mazurkiewicz@outlook.com (R.M.); lukasznawacki@gmail.com (Ł.N.); 2 Independent Public Healthcare Center of the Ministry of Interior and Administration, 80-104 Gdansk, Poland; m.niznik@zozmswia.gda.pl (M.N.); kratnicki@gmail.com (K.R.);; 3 Department of General and Vascular Surgery, Faculty of Medicine, Medical University of Warsaw, 02-091 Warsaw, Poland; piotrmyr@poczta.fm (P.M.); lenarcik@icloud.com (S.L.); 4 Faculty of Medical and Health Sciences, University of Siedlce, 08-110 Siedlce, Poland; chirurgia.siedlce@gmail.com; 5 Siedlce Hospital, 08-110 Siedle, Poland; oklinska.laura@gmail.com (L.K.); pajermalgorzata@gmail.com (M.P.); 6 First Department of General Surgery, University Hospital, 30-688 Cracow, Poland; piotr.richter@uj.edu.pl (P.R.); kamil.rapacz@doctoral.uj.edu.pl (K.R.); 7 Jagiellonian University Medical College, Doctoral School of Medical and Health Sciences, 30-688 Cracow, Poland; 8 Department of General, Minimally Invasive and Endocrine Surgery, Wroclaw Medical University, 50-556 Wroclaw, Poland; maciej.sroczynski@umw.edu.pl (M.S.); mateusz.szmit@umw.edu.pl (M.S.)

**Keywords:** body mass index, metabolic dysfunction-associated steatotic liver disease, obesity, overweight

## Abstract

**Background:** Metabolic dysfunction-associated steatotic liver disease (MASLD) is defined by the presence of hepatic steatosis and at least one cardiometabolic risk factor. Differences in the incidence of MASLD between men and women are primarily due to distinct metabolic and cardiovascular profiles. The aim of this observational study was to investigate the prevalence and characteristics of MASLD in men and women. **Methods:** The study included patients from the Polish Gallstone Surgery Registry diagnosed with MASLD according to current criteria. **Results:** Among 3419 patients, MASLD was diagnosed in 24.2%. Its prevalence was significantly higher in men (29.8%) than in women (21.9%) (*p* < 0.0001), with the highest incidence observed in men aged 70–79 (32.1%) and women aged 70–79 (33.3%). MASLD was associated with overweight in individuals aged < 50 years (OR 1.87; 95% CI: 1.11–3.14; *p* < 0.0186) and in those aged ≥ 50 years (OR 1.99; 95% CI: 1.48–2.68; *p* < 0.001), as well as with obesity in patients aged < 50 years (OR 6.53; 95% CI: 4.08–10.47; *p* < 0.001) and in those aged ≥ 50 years (OR 3.9; 95% CI: 2.92–5.22; *p* < 0.0001). **Conclusions:** In this study, MASLD was diagnosed more frequently in men than in women, and its incidence showed a positive association with increasing BMI. These findings indicate that excess body weight and sex are key predictors of MASLD, highlighting the need for individualized treatment strategies.

## 1. Introduction

Metabolic dysfunction-associated steatotic liver disease (MASLD), formerly known as metabolic dysfunction-associated fatty liver disease (MAFLD) and non-alcoholic fatty liver disease (NAFLD), encompasses a broad spectrum of hepatic and extra-hepatic pathophysiological and clinical manifestations resulting from liver steatosis [1]. According to the current definition, MASLD is diagnosed after excluding causes of steatosis that are not metabolic [2,3].

The prevalence of MASLD is continuously rising, making it the most common liver disease worldwide, affecting approximately 20–30% of adults [4,5]. The risk factors for MASLD are well established; a strong association with obesity, poor nutrition, dyslipidemia, and sedentary lifestyle has been observed. These factors contribute to lipotoxicity, the production of reactive oxygen species, dysbiosis of the intestinal microbiome, genetic predisposition, and the induction of proinflammatory immune mediators [6].

Consequently, patients with MASLD not only present with a worsened cardiometabolic profile [7,8] but are also at a significantly higher risk of death from cardiovascular disease than from liver disease complications [9].

The heterogeneity between females and males in the prevalence, risk factors, and mechanisms of MASLD has long been recognized but remains incompletely understood. Regarding sex-related differences, both the prevalence and severity of MASLD are higher in men than in women of reproductive age. However, after menopause, the condition occurs more frequently in women, suggesting that estrogens have a protective effect [10]. Additionally, estradiol influences fatty acid synthase expression in hepatic and adipose cells, while saturated fatty acids induce endoplasmic reticulum stress and increase mitochondrial free radical generation, leading to cellular injury and liver steatosis [11].

Moreover, sex and age differences are also observed in the prevalence of diabetes mellitus type 2 (T2DM), visceral adipose tissue accumulation, and components of metabolic syndrome—all of these are major risk factors for MASLD [12,13].

There is a lack of evidence exploring sex-related differences in the epidemiological risk factors for MASLD. Therefore, we aimed to investigate the prevalence of MASLD and its characteristics in men and women.

## 2. Materials and Methods

### 2.1. Study Design

This prospective, multicenter, and observational study involved patients from the Polish Gallstone Surgery Registry. Patients who underwent cholecystectomy due to gallstone disease were consecutively enrolled across six surgical hospitals, including four academic centers and two regional hospitals, providing a comprehensive overview of surgical approaches in Poland. The data included patients admitted to one of the participating centers between 2019 and 2022, either for elective or emergency procedures. To ensure an unbiased selection process and create a cohort reflective of real-world conditions, no specific exclusion criteria were applied.

### 2.2. Patient Involvement

Consecutively hospitalized patients who underwent cholecystectomy were included in the study. MASLD was diagnosed based on the current criteria recommended in June 2023 by experts from the following hepatology societies: European Association for the Study of the Liver (EASL), La Asociación Latinoamericana para el Estudio del Hígado (ALEH), and the American Association for the Study of Liver Diseases (AASLD) [10].

The diagnostic criteria for MASLD included the presence of liver steatosis with at least one cardiometabolic factor:

- BMI ≥ 25 kg/m^2^;
- Fasting serum glucose ≥ 5.6 mmol/L (100 mg/dL) or 2 h post-load glucose level ≥ 7.8 mmol/L (≥140 mg/dL) or HbA1c ≥ 5.7% (39 mmol/L) or diabetes mellitus type 2 (T2DM) or treatment for T2DM;
- Blood pressure ≥ 130/85 mmHg or specific antihypertensive drug treatment;
- Plasma triglycerides ≥ 1.7 mmol/L (150 mg/dL) or lipid lowering treatment;
- Plasma high-density lipoprotein cholesterol (HDL-C) ≤ 1.0 mmol/L (40 mg/dL)—men and ≤ 1.3 mmol/L (50 mg/dL)—women or lipid lowering treatment.

In this study, the two latter factors were collectively referred to as atherogenic dyslipidemia.

**Figure 1:**
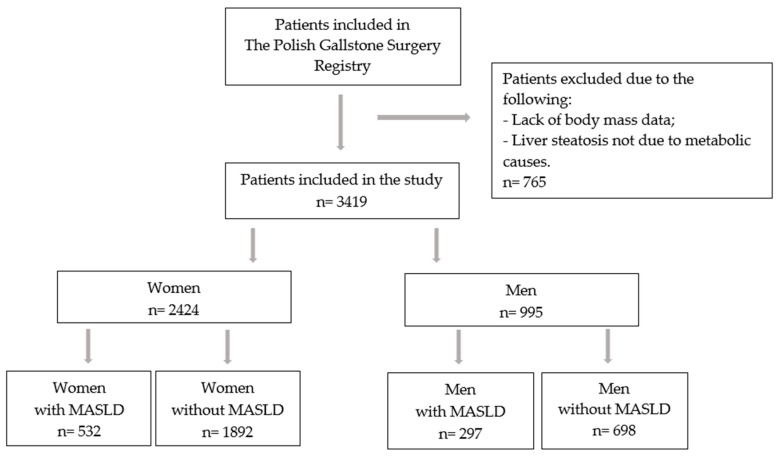
Flow chart of the study. Abbreviation: MASLD—metabolic dysfunction-associated steatotic liver disease.

All the presented diagnostic criteria were available for all the patients included in the study. Patients with incomplete diagnostic data for MASLD, particularly those lacking BMI information, as well as individuals with liver steatosis due to non-metabolic causes (such as alcohol-related or toxic etiologies), were excluded from the analysis (Figure 1).

MASLD was diagnosed during hospitalization. Clinical data regarding comorbidities were obtained through patient history, while laboratory tests and anthropometric measurements were also performed during hospitalization. Hepatic steatosis was assessed using abdominal ultrasonography.

The study was approved by the Ethics Committee of the Collegium Medicum of Jan Kochanowski University (104/2022). This study adhered to the ethical guidelines outlined in the Declaration of Helsinki and its subsequent amendments. The requirement for patient informed consent was waived, as this was a retrospective study conducted exclusively using de-identified data.

### 2.3. Statistical Analyses

Continuous data were described using means and standard deviations, while categorical data were summarized as frequencies and percentages. Group comparisons were conducted using the chi-square or Fisher exact test for categorical variables and *t*-test or Mann–Whitney test for continuous variables. Univariable and multivariable analyses were performed using logistic regression models. Separable multivariable analyses were carried out for younger (<50 years) and older (≥50) patients. For these analyses, odds ratios (OR) with 95% confidence intervals (95% CI) were calculated. A two-tailed *p*-value < 0.05 was considered statistically significant. All the statistical analyses were conducted using the R software package version 4.0.3.

## 3. Results

### 3.1. Patient Characteristics

A total of 3419 participants were analyzed, including 995 men (29.1%) and 2424 women (70.9%). The mean age of the study population was 54.8 years (SD: 15.3). Among the participants, 27 (0.8%) were underweight, 849 (24.8%) had a normal body mass, 1310 (38.3%) were overweight, and 1233 (36.1%) had obesity. Hypertension was diagnosed in 1433 patients (41.9%), T2DM or prediabetes in 519 patients (15.2%), and atherogenic dyslipidemia in 432 patients (12.6%). The baseline characteristics of the study participants categorized by sex are presented in Table 1.

### 3.2. Prevalence of MASLD in Men and Women

Among the patients included in the study, MASLD was diagnosed in 829 patients (24.2%). Its prevalence was significantly higher in the men (n = 297, 29.8%) than in the women (n = 532, 21.9%), *p* < 0.0001. Lean MASLD was diagnosed in 94 patients (11.3% of those with MASLD), including 63 women (8.9% of women without overweight/obesity) and 31 men (17.2% of men without overweight/obesity), *p* = 0.0012. MASLD in the patients with overweight/obesity was diagnosed in 469 women (27.3%) and in 266 men (32.6%), *p* = 0.0057.

### 3.3. Prevalence of Cardiometabolic MASLD Diagnostic Criteria in Men and Women According to BMI

Most patients met two diagnostic criteria for MASLD diagnosis. No significant differences were observed in the number of diagnostic criteria between men and women. Table 2 presents the number of diagnostic criteria by sex.

In the normal body mass group, hypertension, T2DM or prediabetes, and atherogenic dyslipidemia were diagnosed more frequently in men than in women (37.7% vs. 23.4%; 11.2% vs. 5.7%; 14.0% vs. 6.7%, respectively). In contrast, no significant differences in the incidence of these conditions were observed between male and female patients in the overweight group (Table 3).

### 3.4. Comparison of Men and Women with MASLD

In the present study, women and men with MASLD did not differ significantly in age (59.6 vs. 58.5, *p* = 0.2383). The proportion of patients with MASLD and obesity was not statistically higher in women than in men (57.3% vs. 50.5%, *p* = 0.0516). However, gender-related differences were observed across obesity classes. Among the patients with MASLD and obesity, class I obesity was diagnosed in 180 women (58.8%) and 107 men (71.3%)—*p* = 0.0094; class II obesity was diagnosed in 82 women (26.8%) and 27 men (18%)—*p* = 0.0385; and class III obesity was observed in 44 women (14.4%) and 16 men (10.7%)—*p* = 0.2705.

The clinical characteristics of the men and women with MASLD were similar, with no significant differences in the incidence of hypertension, T2DM or prediabetes, atherogenic dyslipidemia, heart failure, or atrial fibrillation (Table 1).

### 3.5. Prevalence of MASLD and Stratification by Age, BMI, and Metabolic Disorder

**Figure 2:**
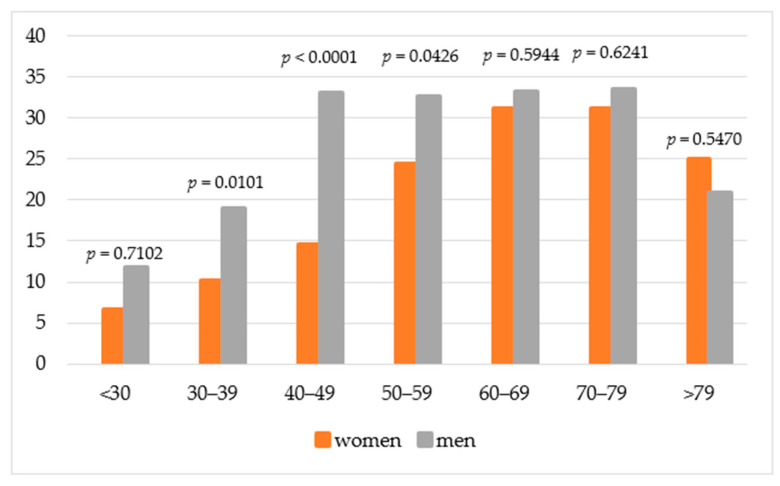
Prevalence of MASLD in different age groups.

The prevalence of MASLD was not linearly associated with age (Figure 2). In the overall population, the prevalence gradually increased with age, peaking at 32.1% in the 70–79 age group. Interestingly, in the men, the prevalence of MASLD rose sharply between the ages of 40–49 and remained stable until age 79, reaching a peak of 33% in the 70–79 age group. In contrast, MASLD prevalence in the women gradually increased in the 50–59 age range, peaking at 33.3% between ages 70 and 79. As expected, the lowest incidence of MASLD in both women and men was observed in the <30 and ≥80 age groups.

The prevalence rates of MASLD in different cardiometabolic groups by sex differed only in the patients with overweight and obesity, with a higher incidence in men than in women (32.6% vs. 27.2%, *p* = 0.0057). In the patients with hypertension, those with T2DM or prediabetes, and those with atherogenic dyslipidemia, the prevalence of MASLD did not differ between the women and the men (Table 4).

### 3.6. Risk Factors for MASLD

The relationship between MASLD, age, and BMI is presented in Table 5, which shows the incidence of MASLD across the BMI categories and various age groups in both the females and the males.

Multivariable logistic regression analysis showed that younger age group was associated with MASLD incidence. As expected, the risk increased with an increase in BMI. In patients with BMI ≥ 30 kg/m^2^, the risk was significantly high in younger and older patients.

## 4. Discussion

The prevalence of MASLD appears to differ between men and women depending on age and the presence of comorbidities. The major findings of this study are as follows. Firstly, our country-specific registry data revealed that the incidence of MASLD is higher in men than in women. Secondly, the incidence of MASLD increases with age and in both sexes, reaching a peak between 70 and 79 years. Thirdly, higher body weight was identified as a strong risk factor for MASLD, independent of age.

Our findings indicate that MASLD posed a significant burden on the population undergoing cholecystectomy, with an overall prevalence of 24.2%. Although the study population consisted of patients with gallstone disease, the incidence of MASLD was similar to that in the general population. Notably, we observed a sex-specific difference in MASLD prevalence, with men having a higher prevalence (29.8%) than women (21.9%), suggesting that men are more susceptible to MASLD than women. The higher prevalence of MASLD in men may increase their vulnerability to chronic diseases such as hypertension, cardiovascular disease, T2DM, and stroke.

It should be emphasized that currently there is evidence suggesting a potential association between gallstone disease and MASLD. Both gallstone disease and MASLD represent increasingly prevalent global pathologies contributing to a substantial economic and healthcare burden [14,15]. However, the underlying etiology linking these two conditions remains unclear [16,17]. Even among individuals with normal BMI, patients with MASLD have a 1.29-fold higher risk of gallstone disease [18]. Furthermore, gallstone disease incidence is influenced not only by sex and BMI but also by age, waist circumference, and smoking habits, which indicates that the development of gallstone disease and MASLD originate from similar risk factors [19,20].

Crudele et al. [21] reported that MASLD was diagnosed in 59% of patients having metabolic syndrome, with a higher incidence observed in men. A Spanish study found the overall prevalence of NAFLD to be 19.1%, with rates of 27.9% in men and 6.8% in women, increasing across age groups [22]. Data from the National Health and Nutrition Examination Survey III (NHANES III) also indicated that MASLD is more common in men than in women. Similarly, Chang et al. [23] demonstrated a clear male predominance in MASLD prevalence, with a higher rate in men (45.7%) than in women (23.9%). It is important to note that MASLD is more frequently observed in Asian populations [24,25]. However, another study reported a higher prevalence in women (31.7%) compared to men (25.5%), which may be attributed to a smaller sample size and, more significantly, differences in the age distribution of the study population [26]. That study included patients with a mean age of 67 years, whereas the mean age in our study was 55.

The inclusion of a higher proportion of postmenopausal women may have influenced the results, as estrogen has been shown to have a protective effect against MASLD [27,28,29]. Studies on sex differences in MASLD show that MASLD is more prevalent in men than in women of reproductive age. However, after menopause, this difference diminishes, exposing older women to higher rates of MASLD. Analysis of the Polish Gallstone Surgery Registry showed that the incidence of MASLD increased with age, with the highest percentage of patients observed in the 70–79 age group for both women and men. Chang et al. [23] reported the highest prevalence of MASLD in the 50–59 age group, suggesting that it is more common in middle-aged individuals. A high proportion of MASLD diagnoses in men was observed between 40 and 49 years, whereas in women it was most common between 50 and 59 years. This difference may be influenced by hormonal changes in women, making them more susceptible to MASLD at an older age. Conversely, younger men have a higher incidence of metabolic and cardiovascular diseases, which may predispose them to develop MASLD at an earlier age than women.

Interestingly, in the present study, the incidence of MASLD did not differ between men and women in the subgroups of patients having hypertension, T2DM/prediabetes, or atherogenic dyslipidemia. Numerous epidemiological studies have shown that overweight and obesity are significant, independent, and modifiable risk factors for MASLD, as evidenced by the parallel rise in obesity rates and prevalence of MASLD [7,30,31]. Chen et al. [25] also identified a strong, nearly linear relationship between MASLD incidence and increased BMI. Regardless of other metabolic abnormalities, patients with obesity face a higher risk of developing MASLD. Accordingly, the prevention of MASLD requires effective strategies targeting both the prevention and treatment of obesity. The data regarding the role of excess body weight in the development of MASLD is unequivocal and it is well established that obesity most frequently results from insufficient physical activity combined with unhealthy dietary habits. Current clinical guidelines for the management of patients with MASLD highlight the central role of non-pharmacological interventions, which are considered fundamental both in the prevention and in the therapeutic management of this condition [32,33].

However, our subgroup analysis of age and BMI revealed distinct sex differences, showing that men are more prone to MASLD than women, particularly at a younger age. Furthermore, our data demonstrated a significant link between MASLD, BMI, and age, with higher BMI and younger age strongly associated with greater prevalence. These findings suggest that elevated BMI, especially in younger men, may be a key factor in the onset and progression of MASLD.

## 5. Strengths and Limitations

The present study has several strengths, including the focus on MASLD as a common and highly relevant clinical problem, the use of data obtained from a multicenter study, and the consideration of sex-related differences in the epidemiology of metabolic diseases, which are of particular importance when planning preventive and early diagnostic strategies tailored to women and/or men. It should also be emphasized that in recent years the definition and diagnostic criteria of MASLD have undergone dynamic changes, further highlighting the importance of continuously addressing this condition.

This study has inherent limitations, the primary one being the specific population analyzed—patients who underwent cholecystectomy for gallstone disease. This limitation may be considered from two perspectives. On the one hand, the findings may not be fully generalizable to a broader population, even though the incidence of MASLD observed in this study is consistent with that reported in the general population. On the other hand, it should be noted that the study cohort displayed a prevalence of MASLD, including sex-related distribution between women and men, similar to that observed in the general population. Additionally, it is important to recognize that the study cohort predominantly consisted of women, which may have influenced the results. As with all retrospective studies, the presence of unidentified confounders cannot be excluded. In particular, we were unable to adjust for individual-level socioeconomic status, duration of diabetes mellitus, or hormonal status within the study group. Furthermore, another limitation is the incomplete availability of certain data, as some variables were missing for some patients (e.g., laboratory tests other than those required for the diagnosis of MASLD).

## 6. Conclusions

Our results suggest a high prevalence of MASLD and other metabolic conditions, particularly obesity, in patients undergoing cholecystectomies. In both men and women, the prevalence of MASLD increased with age and BMI. Prioritizing the prevention and management of metabolic disorders, especially obesity, is crucial for the effective treatment of MASLD. These findings underscore the importance of developing personalized interventions to prevent and manage MASLD in both women and men.

## Figures and Tables

**Table 1:** Comparison of patients with and without MASLD categorized by sex.

Clinical Characteristic	Women n = 2424			Men n = 995			*p* MASLD Women vs. MASLD Men
	MASLD n = 532	Non-MASLD n = 1892	*p*	MASLD n = 297	Non-MASLD n = 698	*p*	
Age, years, mean (SD)	59.6 (13.0)	52.4 (15.5)	<0.0001	58.5 (13.2)	56.1 (15.6)	0.0150	0.2383
BMI, kg/m^2^							
<18.5	1 (0.2)	23 (1.2)	<0.0001	0 (0.0)	3 (0.4)	<0.0001	0.0762
18.5–24.9	61 (11.5)	613 (32.4)		31 (10.4)	144 (20.6)		
25.0–29.9	164 (30.8)	686 (36.3)		116 (39.1)	344 (49.3)		
≥30	306 (57.5)	570 (30.1)		150 (50.5)	207 (29.7)		
BMI, kg/m^2^, mean (SD)	31.5 (5.7)	27.7 (5.2)	<0.0001	30.4 (4.8)	28.1 (4.3)	<0.0001	0.0032
Comorbidities							
Hypertension	292 (54.9)	662 (35)	<0.0001	169 (56.9)	310 (44.4)	0.0003	0.5755
T2DM	115 (21.6)	153 (8.1)	<0.0001	60 (20.2)	87 (12.5)	0.0016	0.6323
Prediabetes	29 (5.5)	59 (3.1)	0.0110	7 (2.4)	9 (1.3)	0.2697	0.0361
T2DM or prediabetes	144 (27.1)	212 (11.2)	<0.0001	67 (22.6)	96 (13.8)	0.0006	0.1530
Atherogenic dyslipidemia	255 (47.9)	30 (1.6)	<0.0001	125 (42.1)	22 (3.2)	<0.0001	0.1054
Chronic coronary syndrome	29 (5.5)	88 (4.7)	0.4469	30 (10.1)	57 (8.2)	0.3228	0.0125
Previous myocardial infarction	13 (2.4)	32 (1.7)	0.2561	16 (5.4)	26 (3.7)	0.2328	0.0270
Hypercholesterolemia	80 (15)	135 (7.1)	<0.0001	39 (13.1)	48 (6.9)	0.0014	0.4529
Atrial fibrillation	21 (3.9)	47 (2.5)	0.0710	20 (6.7)	35 (5)	0.2774	0.0760
Thromboembolism events	7 (1.3)	18 (1)	0.4623	15 (5.1)	25 (3.6)	0.2804	0.0013
Venous thromboembolism	14 (2.6)	31 (1.4)	0.1343	10 (3.4)	20 (2.9)	0.6720	0.5448
Heart failure	12 (2.3)	41 (2.2)	0.9017	14 (4.7)	27 (3.9)	0.5392	0.0515
Acute pancreatitis previous	29 (5.5)	76 (4.0)	0.1511	16 (5.4)	43 (6.2)	0.6365	0.9689
Chronic pancreatitis	3 (0.6)	9 (0.5)	0.7328	2 (0.7)	5 (0.7)	1	1
Malignancy	47 (8.8)	113 (6)	0.0188	16 (5.4)	47 (6.7)	0.4249	0.0725

Data are presented as number (percentage) unless otherwise indicated. Abbreviations: BMI—body mass index; MASLD—metabolic dysfunction-associated steatotic liver disease; T2DM—diabetes mellitus type 2.

**Table 2:** Number of factors included in MASLD diagnosis.

Criteria of MASLD Diagnosis	All MASLD n = 829	Women with MASLD n = 532	Men with MASLD n = 297	*p*
1 criterion	162 (19.5)	99 (18.6)	63 (21.2)	0.3648
2 criteria	493 (59.5)	318 (59.8)	175 (58.9)	0.8107
3 criteria	147 (17.7)	98 (18.4)	49 (16.5)	0.4871
4 criteria	27 (3.3)	17 (3.2)	10 (3.4)	0.8939

Data are presented as number values (percentages). Abbreviations: MASLD—metabolic dysfunction-associated steatotic liver disease.

**Table 3:** Prevalence of MASLD and cardiometabolic conditions in various BMI groups among women and men.

	BMI ≤ 24.9 kg/m^2^ n = 876			BMI 25–29.9 kg/m^2^ n = 1310			BMI ≥ 30 kg/m^2^ n = 1233		
	Women n = 698	Men n = 178	*p*	Women n = 850	Men n = 460	*p*	Women n = 876	Men n = 357	*p*
MASLD	62 (8.9)	31 (17.4)	0.0010	164 (19.3)	116 (25.2)	0.0126	306 (34.9)	150 (42.0)	0.0194
Hypertension	163 (23.4)	67 (37.6)	0.0001	353 (41.5)	198 (43.0)	0.5962	438 (50.0)	214 (59.9)	0.0015
T2DM or prediabetes	40 (5.7)	20 (11.2)	0.0094	117 (13.8)	57 (12.4)	0.4845	199 (22.7)	86 (24.1)	0.6040
Atherogenic dyslipidemia	47 (6.7)	25 (14.0)	0.0015	61 (7.2)	32 (7.0)	0.8824	177 (20.2)	90 (25.2)	0.0530

Data are presented as number values (percentages). Abbreviations: BMI—body mass index; MASLD—metabolic dysfunction-associated steatotic liver disease; T2DM—diabetes mellitus type 2.

**Table 4:** Prevalence of MASLD across different cardiometabolic groups in women and men.

	Overweight or Obesity n = 2543	Hypertension n = 1433	T2DM or Prediabetes n = 519	Atherogenic Dyslipidemia n = 432
All	736 (28.9)	461 (32.2)	211 (40.7)	380 (88)
Women	470/1726 (27.2)	292/954 (30.6)	144/356 (40.4)	255/285 (89.5)
Men	266/817 (32.6)	169/479 (35.3)	67/163 (41.1)	125/147 (85)
*p* value	0.0057	0.0740	0.8879	0.1791

Data are presented as number values (percentages). Abbreviations: BMI—body mass index; MASLD—metabolic dysfunction-associated steatotic liver disease; T2DM—diabetes mellitus type 2.

**Table 5:** Multivariable logistic regression analysis including age, sex, and BMI as predictors of MASLD.

		Age < 50 Years			Age ≥ 50 Years		
		Multivariable OR	95% CI	*p*	Multivariable OR	95% CI	*p*
Gender	Female	Ref.			Ref.		
	Male	2.55	1.83–3.56	<0.0001	1.14	0.93–1.4	0.2146
BMI kg/m^2^	<25	Ref.			Ref.		
	25.0–29.9	1.87	1.11–3.14	<0.0186	1.99	1.48–2.68	<0.001
	≥30	6.53	4.08–10.47	<0.0001	3.90	2.92–5.22	<0.0001

Abbreviations: BMI—body mass index; CI—confidence interval; OR—odds ratio.

## Data Availability

The original contributions presented in this study are included in the article. Further inquiries can be directed to the corresponding author.

## References

[1] Rinella M.E., Lazarus J.V., Ratziu V., Francque S.M., Sanyal A.J., Kanwal F., Romero D., Abdelmalek M.F., Anstee Q.M., Arab J.P. (2023). NAFLD Nomenclature consensus group. A multi-society Delphi consensus statement on new fatty liver disease nomenclature. J. Hepatol..

[2] Janczura J., Brzdęk M., Dobrowolska K., Flisiak R., Martonik D., Brzdęk K., Pleśniak R., Kukla-Woźnica D., Wajdowicz M., Zarębska-Michaluk D. (2025). Steatotic liver disease in patients treated for chronic hepatitis B. Pol. Arch. Intern. Med..

[3] Dixon W., Corey K.E., Luther J., Goodman R.P., Schaefer E.A. (2025). Prevalence and Clinical Correlation of Cardiometabolic Risk Factors in Alcohol-Related Liver Disease and Metabolic Dysfunction and Alcohol Associated Liver Disease (MetALD). J. Clin. Exp. Hepatol..

[4] Pimpin L., Cortez-Pinto H., Negro F., Corbould E., Lazarus J.V., Webber L., Sheron N. (2018). Burden of liver disease in Europe: Epidemiology and analysis of risk factors to identify prevention policies. J. Hepatol..

[5] Yang A.H., Tincopa M.A., Tavaglione F., Ajmera V.H., Richards L.M., Amangurbanova M., Butcher C., Hernandez C., Madamba E., Singh S. (2024). Prevalence of steatotic liver disease, advanced fibrosis and cirrhosis among community-dwelling overweight and obese individuals in the USA. Gut.

[6] Hong S., Sun L., Hao Y., Li P., Zhou Y., Liang X., Hu J., Wei H. (2024). From NAFLD to MASLD: When metabolic comorbidity matters. Ann. Hepatol..

[7] Zhao D., Zheng X., Wang L., Xie Y., Chen Y., Zhang Y. (2025). Overlap prevalence and interaction effect of cardiometabolic risk factors for metabolic dysfunction-associated steatotic liver disease. Nutr. Metab..

[8] Stefan N., Yki-Järvinen H., Neuschwander-Tetri B.A. (2025). Metabolic dysfunction-associated steatotic liver disease: Heterogeneous pathomechanisms and effectiveness of metabolism-based treatment. Lancet Diabetes Endocrinol..

[9] Mayén A.L., Sabra M., Aglago E.K., Perlemuter G., Voican C., Ramos I., Debras C., Blanco J., Viallon V., Ferrari P. (2024). Hepatic steatosis, metabolic dysfunction and risk of mortality: Findings from a multinational prospective cohort study. BMC Med..

[10] Lonardo A., Nascimbeni F., Ballestri S., Fairweather D., Win S., Than T.A., Abdelmalek M.F., Suzuki A. (2019). Sex differences in nonalcoholic fatty liver disease: State of the art and identification of research gaps. Hepatology..

[11] Chen K.L., Madak-Erdogan Z. (2018). Estrogens and female liver health. Steroids..

[12] Colosimo S., Mitra S.K., Chaudhury T., Marchesini G. (2023). Insulin resistance and metabolic flexibility as drivers of liver and cardiac disease in T2DM. Diabetes Res. Clin. Pract..

[13] Qureshi K., Abrams G.A. (2007). Metabolic liver disease of obesity and role of adipose tissue in the pathogenesis of nonalcoholic fatty liver disease. World J. Gastroenterol..

[14] Wang X., Yu W., Jiang G., Li H., Li S., Xie L., Bai X., Cui P., Chen Q., Lou Y. (2024). Global Epidemiology of Gallstones in the 21st Century: A Systematic Review and Meta-Analysis. Clin. Gastroenterol. Hepatol..

[15] Kim Y., Rydqvist P., Ramezani T., Haas J.S., Bantel H., Buggisch P., Geier A., Hofmann W.P., Mauss S., Roeb E. (2025). Metabolic Dysfunction-Associated Steatohepatitis Diagnosis and Management in Germany: Insights From an Expert Consensus Panel. Liver Int..

[16] Roesch-Dietlen F., Pérez-Morales A.G., Grube-Pagola P., González-Santes M., Díaz-Roesch F., Triana-Romero A., Roesch-Ramos L., Remes-Troche J.M., Cruz-Aguilar M. (2023). Prevalence of metabolic associated fatty liver disease (MAFLD) in patients with gallstone disease. Study on a cohort of cases in South-Southeastern Mexico. Rev. Gastroenterol. Mex..

[17] Zhao G., Shi R., Ma M., Lin H., Zhang J., Sheng B. (2024). Elevated LDL-c may warn of the risk of gallbladder stones in the patients with metabolic dysfunction-associated steatotic liver disease: A case-control study. Clin. Res. Hepatol. Gastroenterol..

[18] Kim N.H., Kang J.H., Kim H.J. (2024). Association between nonalcoholic fatty liver disease and gallstone risk in nonobese and lean individuals. Eur. J. Gastroenterol. Hepatol..

[19] Shabanzadeh D.M., Holmboe S.A., Sørensen L.T., Linneberg A., Andersson A.M., Jørgensen T. (2017). Are incident gallstones associated to sex-dependent changes with age? A cohort study. Andrology.

[20] Sogabe M., Okahisa T., Kagawa M., Kashihara T., Fujmoto S., Kawaguchi T., Yokoyama R., Kagemoto K., Tanaka H., Kida Y. (2024). Association of metabolic dysfunction-associated fatty liver disease with gallstone development: A longitudinal study. J. Gastroenterol. Hepatol..

[21] Najafi F., Pasdar Y., Nazar M.M., Darbandi M. (2024). Association between obesity phenotypes and non-alcoholic fatty liver: A large population-based study. BMC Endocr. Disord..

[22] Fresneda S., Abbate M., Busquets-Cortés C., López-González A., Fuster-Parra P., Bennasar-Veny M., Yáñez A.M. (2022). Sex and age differences in the association of fatty liver index-defined non-alcoholic fatty liver disease with cardiometabolic risk factors: A cross-sectional study. Biol. Sex. Differ..

[23] Ji H., Cheng S. (2024). Heart-Liver Axis Research Collaboration: Sex differences in prevalence and prognosis of steatotic liver disease phenotypes: Biological sex matters. J. Hepatol..

[24] Chang M., Shao Z., Wei W., Shen P., Shen G. (2023). Sex-specific prevalence and risk factors of metabolic-associated fatty liver disease among 75,570 individuals in eastern China. Front. Endocrinol..

[25] Chen Y.L., Li H., Li S., Xu Z., Tian S., Wu J., Liang X.Y., Li X., Liu Z.L., Xiao J. (2021). Prevalence of and risk factors for metabolic associated fatty liver disease in an urban population in China: A cross-sectional comparative study. BMC Gastroenterol..

[26] Lei F., Qin J.J., Song X., Liu Y.M., Chen M.M., Sun T., Huang X., Deng K.Q., Zuo X., Yao D. (2022). The prevalence of MAFLD and its association with atrial fibrillation in a nationwide health check-up population in China. Front. Endocrinol..

[27] Duan H., Gong M., Yuan G., Wang Z. (2025). Sex Hormone: A Potential Target at Treating Female Metabolic Dysfunction-Associated Steatotic Liver Disease?. J. Clin. Exp. Hepatol..

[28] Alves E.S., Santos J.D.M., Cruz A.G., Camargo F.N., Talarico C.H.Z., Santos A.R.M., Silva C.A.A., Morgan H.J.N., Matos S.L., Araujo L.C.C. (2025). Hepatic Estrogen Receptor Alpha Overexpression Protects Against Hepatic Insulin Resistance and MASLD. Pathophysiology.

[29] Cherubini A., Della Torre S., Pelusi S., Valenti L. (2024). Sexual dimorphism of metabolic dysfunction-associated steatotic liver disease. Trends. Mol. Med..

[30] Ma X.M., Guo Y.M., Jiang S.Y., Li K.X., Zheng Y.F., Guo X.G., Ren Z.Y. (2025). Potential predictive role of Non-HDL to HDL Cholesterol Ratio (NHHR) in MASLD: Focus on obese and type 2 diabetic populations. BMC Gastroenterol..

[31] Shi S., Zhou F., Shen J. (2025). Trends in the prevalence of cardiometabolic diseases in US adults with newly diagnosed and undiagnosed diabetes, 1988–2020. Public Health.

[32] Romeo S., Vidal-Puig A., Husain M., Ahima R., Arca M., Bhatt D.L., Diehl A.M., Fontana L., Foo R., Frühbeck G. (2025). Clinical staging to guide management of metabolic disorders and their sequelae: A European Atherosclerosis Society consensus statement. Eur. Heart J..

[33] European Association for the Study of the Liver (EASL), European Association for the Study of Diabetes (EASD), European Association for the Study of Obesity (EASO) (2024). EASL-EASD-EASO Clinical Practice Guidelines on the management of metabolic dysfunction-associated steatotic liver disease (MASLD). J. Hepatol..

